# Intraflagellar transport 46 (IFT46) is essential for trafficking IFT proteins between cilia and cytoplasm in *Paramecium*

**DOI:** 10.1038/s41598-018-27050-8

**Published:** 2018-06-18

**Authors:** Lei Shi, Xuezhen Shi, Yuan Shen

**Affiliations:** 1 School of Basic Medical Sciences, Xinxiang Medical University, Xinxiang, 453003 P.R. China; 2 School of Life Science, Henan Normal University, Xinxiang, 453007 P.R. China; 3 School of Pharmacy, Xinxiang Medical University, Xinxiang, 453003 P.R. China

## Abstract

Intraflagellar transport (IFT) is a bi-directional process by which particles are carried within the cilia or flagella. This process is essential for ciliary growth and functional maintenance. The IFT complex B (IFTB) is linked to a kinesin motor for anterograde transport towards the ciliary tip. The IFT complex A (IFTA) is connected to a dynein motor for retrograde transport towards the ciliary basis. This study focuses on IFT46, an IFTB member that participates in this process. In *Paramecium*, a GFP-labelled IFT46 protein was found in basal bodies and in some cilia, mostly those undergoing biogenesis. RNA interference against IFT46 in *Paramecium* triggered severe defects in ciliary growth and architecture, including a decreased cilia number and shortened cilia length. This result differed from that obtained from the cells that were depleted of IFT80, another IFTB protein. Moreover, IFT57-GFP fusion protein abnormally accumulated in the cortex and cytoplasm in IFT46-depleted cells compared with the control. Furthermore, transcriptomic analysis showed that IFT46 depletion induced the abnormal expression of several genes that encodeding kinesin and dynein chains. These findings together indicate that IFT46 plays important roles in trafficking IFT proteins between the cytoplasm and cilia of *Paramecium*.

## Introduction

Cilia or flagella are conserved eukaryotic organelles that protrude from the cell surface. The microtubular backbone axoneme of these organelles is anchored at a centriolar structure called the basal body. In addition to motility organelles, cilia and flagella are involved in sensory functions. Almost all cell types in mammals are ciliated, with at least a single primary cilium acting as a cell antenna in intercellular signalling^1,2^. Structural and functional defects of primary cilia induce a wide range of human disorders, which are termed ciliopathies^3^. The biogenesis of cilia involves intraflagellar transport (IFT), a specific movement mechanism that was first discovered in *Chlamydomonas*^4–6^. Generally, the IFT complex is composed of subcomplex A and subcomplex B, each of which contain at least 22 IFT members^7,8^. The IFT subcomplex B (IFTB) is linked to a kinesin II motor for anterograde transport towards the ciliary tip during cilia building, whereas the IFT subcomplex A (IFTA) is connected to a dynein motor for retrograde transport towards the ciliary basis during cilia recycling.

In most species, IFT proteins are essential for cilia growth and ciliary structure. IFTB mutations often cause defects in ciliogenesis, and the absence of IFTA can induce an abnormal accumulation of other IFT proteins at the cilia tip. However, some recently discovered roles of IFTA and IFTB proteins are not limited to cilia. For example, IFT20 has important functions in the mammalian immune synapse^9,10^. IFT88 is required for the orientation of the mitotic apparatus^11,12^, a complex of IFT27 and IFT25 is important for male fertility^13^ and our previous study in Paramecium, a unicellular model with thousands of motile cilia, also showed that IFT57 plays dual roles in cilia and the macronucleus^14^. IFTA protein also shows their functions besides cilia, IFT140 is essential for male fertility and dentinogenesis^15,16^, and IFT122 is important for the transportation of opsin in photoreceptor^17^.

IFT46 is a core component of IFT complex B. It binds IFT52 and IFT88 to form a stable trimetric complex^18^. Research on *Chlamydomonas reinhardtii*, *Caenorhabditis elegans* and mammals showed that IFT46 is essential for cilia assembly and vertebrate development^19,20^. In addition, a study on *C*. *reinhardtii* revealed that IFT46 is specifically required to transport outer dynein arms (ODAs) into the flagella^21^. Another study in mice showed that IFT46 stimulates selective gene expression in mammals^22^.

In the present study, we examined the characteristic function and distribution of IFT46 in the cilia, cortex and cytoplasm of *Paramecium*. We found that RNA interference (RNAi) against IFT46 inhibited ciliogenesis, which caused severe ciliary structure defects in *Paramecium*. At the same time, we knocked down IFT80, another IFTB protein in *Paramecium*, by using RNAi. The inhibition of IFT80 disturbed ciliary growth but rarely affected the length of existing cilia, which differed from the result obtained from IFT46 knockdown. Moreover, we confirmed that the depletion of IFT46 induced an abnormal accumulation of other IFTB-GFP fusion proteins in the cortex and cytoplasm of *Paramecium*. Then, a transcriptomic analysis was performed to detect and classify the putative target genes that were specifically regulated by IFT46. All these results in *Paramecium* suggest that IFT46 is essential for cilia assembly and plays important roles in the transport of IFT proteins between the cytoplasm and cilia.

## Results

### Depletion of IFT46 vs. IFT80 in *Paramecium* causes different cilia defects

A unique IFT46 gene was first identified in the *P*. *tetraurelia* genome from ParameciumDB by BLAST. This gene encodes one 289-amino-acid protein that contains a predictable IFTB protein 46 C-terminal domain (IFT46_B_C). The result of multiple alignments between 19 organisms showed that *Paramecium* IFT46 protein shared a high level of amino acid sequence similarity in the IFT46_B_C domain to its counterparts in most eukaryotes (Fig. S1).

At the beginning of our study, the putative function of IFT46 in *Paramecium* was detected by knocking it down with RNAi. After analysing the IFT46 gene sequence, we constructed a vector containing the designed RNAi target gene sequence and transformed it into *Escherichia coli* HT115. The double-stranded RNA produced from the vector down-regulated the expression of IFT46 in *Paramecium* via an RNA homolog-dependent gene-silencing mechanism (see Materials and Methods). Related phenotypes were observed and recorded through microscopy in each round of experiments (n > 5).

**Figure 1:**
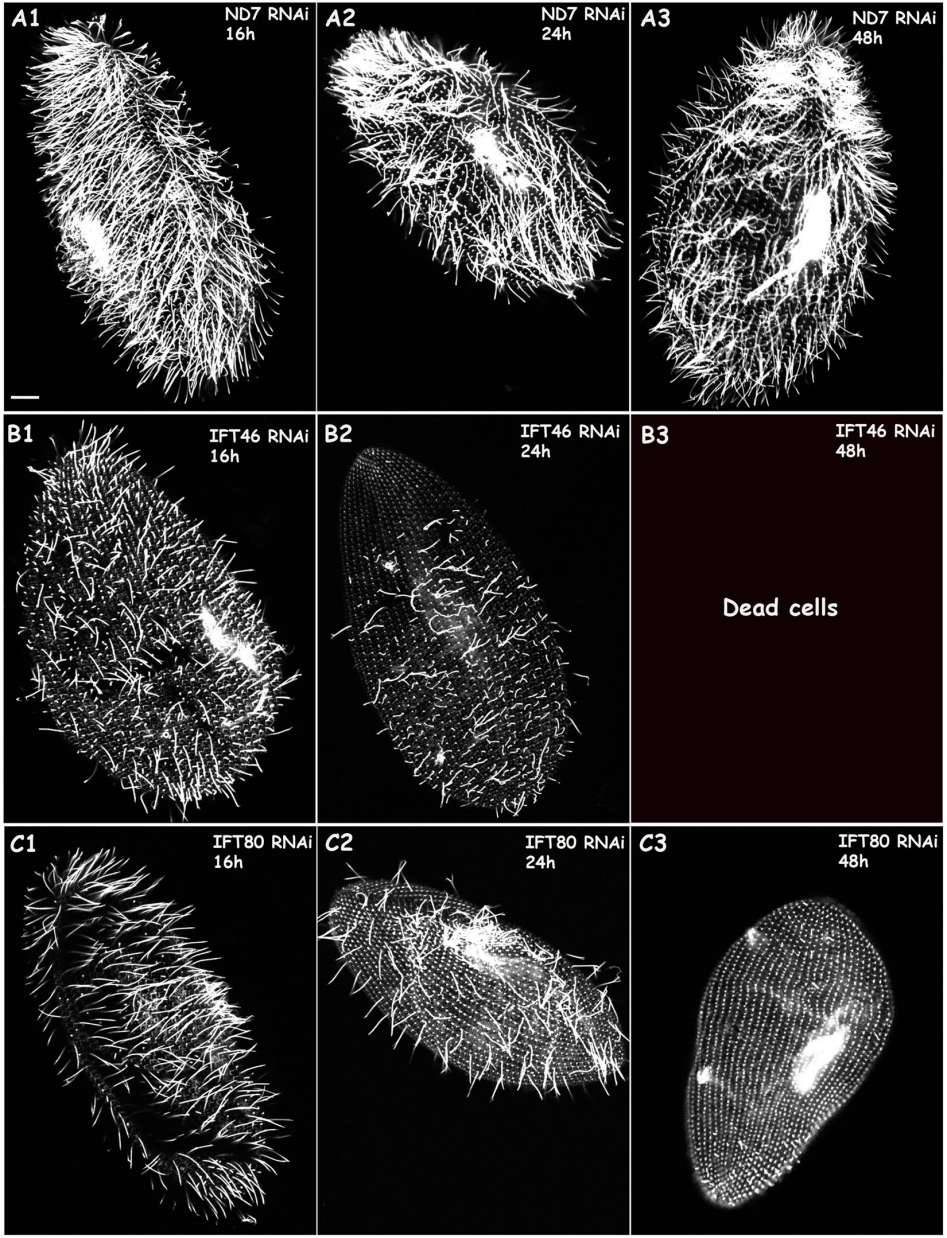
Cilia defects caused by the depletion of IFT46 and IFT80 in *Paramecium*. Immunostaining of cells with anti-tubulin showed ciliary structures when wild-type *Paramecium* cells were treated with ND7 RNAi (control), IFT46 RNAi or IFT80 RNAi. (**A1**–**A3**) Control ND7 RNAi cells displayed a normal (wild-type) number and length of cilia after 16, 24, or 48 h of feeding. (**B1**–**B3**) Cells that were depleted of IFT46 showed a dramatic decrease in cilia division number after 16 or 24 h of feeding, whereas various lengths of cilia emerged on the cells treated with RNAi for 16 or 24 h, and no clones survived after 48 h of feeding, as shown in (**B3**). (**C1**–**C3**) *Paramecium* cells treated with IFT80 RNAi showed cilia loss after 16, 24, or 48 h of RNAi; however, cells with IFT80 knockdown showed mostly normal-length and some extremely short cilia after 16 or 24 h of feeding. Cells that were fed after 48 h lost almost all cilia, as shown in (**C3**). Bar: 10 µm.

**Figure 2:**
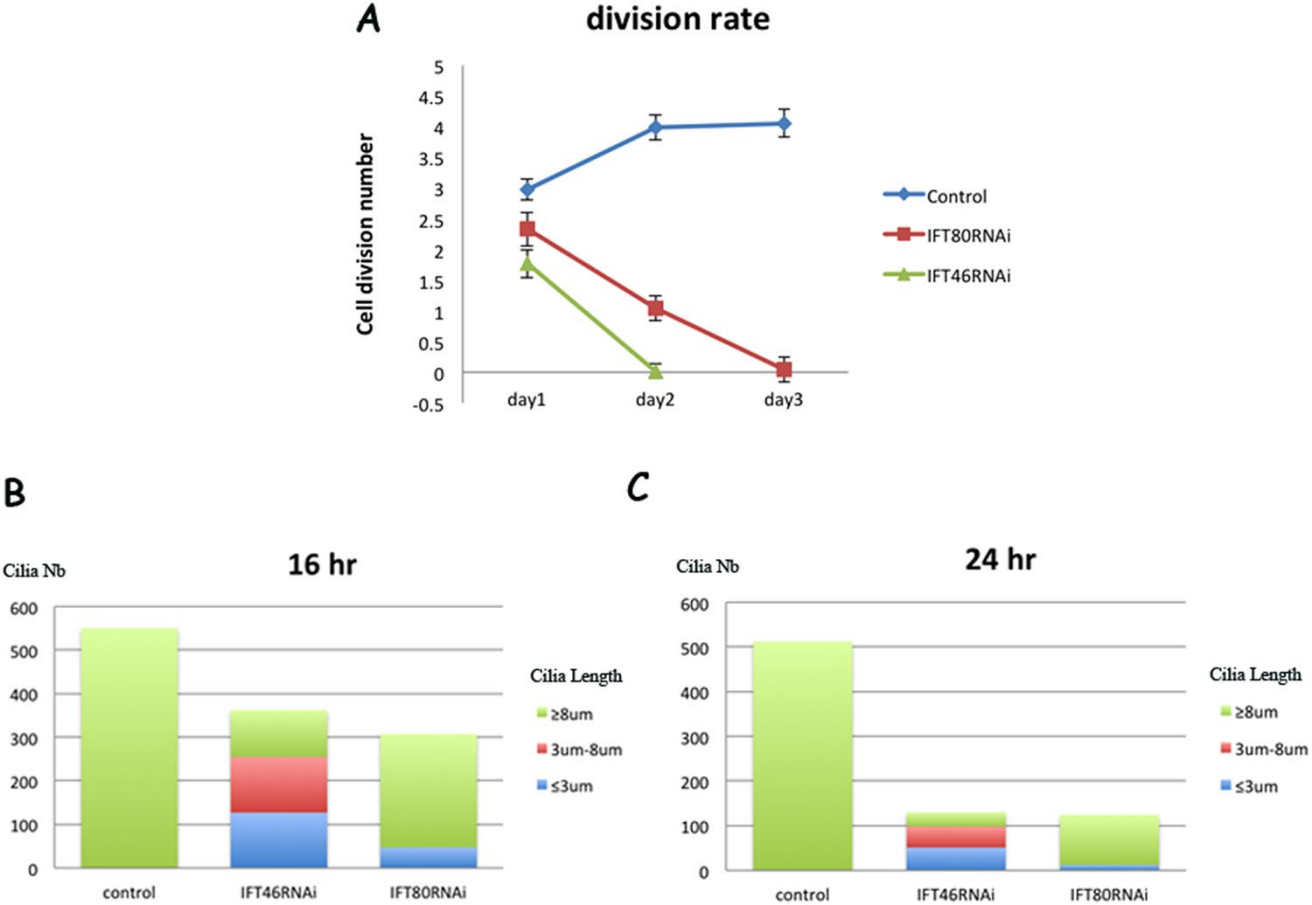
Analysis of cilia defects caused by the knockdown of IFT46 and IFT80 in *Paramecium*. (**A**) The three coloured lines show different division rates after wild-type *Paramecium* cells were fed on Day 1, Day 2 and Day 3. The blue line represents the division rate of ND7 RNAi clones as control, the red line represents the division rate of IFT80 RNAi clones, and the green line represents the division rate of IFT46 RNAi clones. (**B**,**C**) These two graphs describe the changes in length and number of cilia after wild-type *Paramecium* cells were treated with different RNAi for 16 or 24 h.

Using immunofluorescence, we checked the presence of cilia in the RNAi and control groups. The depletion of IFT46 or IFT80 after feeding for 16 h induced fewer cilia than there were in the control (decreases of 29.01% for IFT46 and 39.81% for IFT80). Interestingly, the cells that were depleted of IFT80 had mostly normal-length cilia (longer than 8 μm) and a few extremely short cilia (less than 3 μm) (Figs 1 and 2). In contrast, the IFT46 RNAi cells had mostly cilia between 3 and 8 μm. After RNAi feeding for 24 h, the cells that were silenced using IFT46 lost most of their cilia (a decrease of 76.36%), whereas most of the remaining cilia presented various lengths between 3 and 8 μm. Meanwhile, IFT80 RNAi cells showed fewer cilia (decreased by approximately 78.91%), and most leaving cilia had a normal length (longer than 8 μm). On the third day after RNAi feeding, IFT80 RNAi cells had lost almost all of their cilia, and no IFT46 RNAi cells had survived.

We also recorded the fission times for every day after the cells were treated with RNAi. When the IFT46 gene products were knocked down in *Paramecium*, cells underwent one or two divisions on the first day and then died at a high rate, in contrast to an average of four fissions per day followed by healthy growth in the control ND7 RNAi cells. At the same time, the knockdown of IFT80, another IFTB gene, resulted in a growth rate of two or three divisions on the first day, with a significant decrease in swimming speed and a high rate of death after feeding for 48 h (Fig. 2).

**Figure 3:**
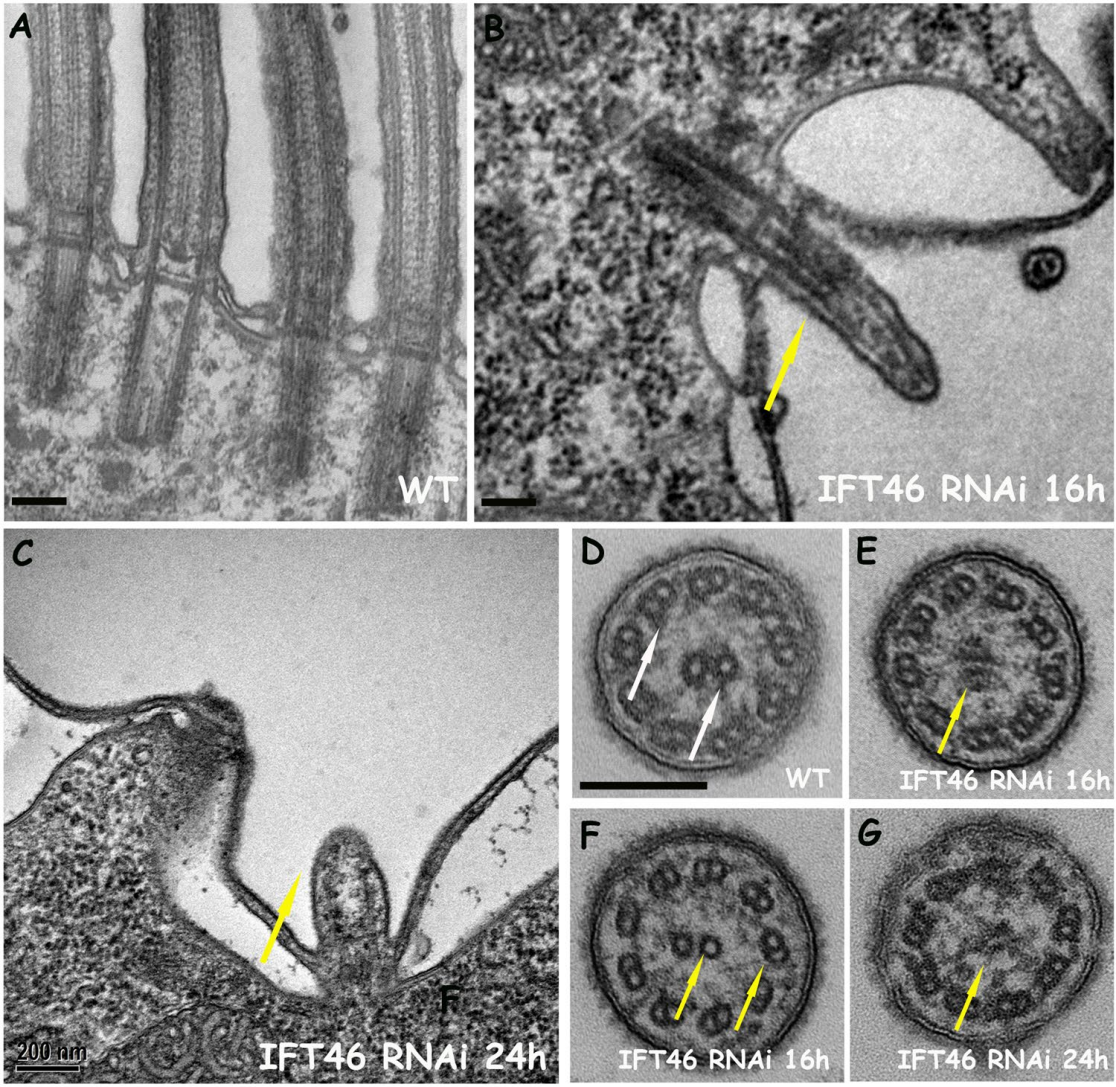
EM photos show defects of the ciliary structure by the depletion of IFT46 in *Paramecium*. Photos (**A**–**G**) show electron micrographs of sections of cilia in wild-type (WT) cells or in IFT46 RNAi cells after 16 or 24 h of feeding. (**A**,**D**) Wild-type. (**B**,**E**,**F**) IFT46 RNAi after 16 h. (**C**,**G**) IFT46 RNAi after 24 h. Yellow arrows show short cilia with ill-organised microtubules. Bar: 200 nm.

Photos captured under an electron microscope (EM) showed the presence of the microtubular backbone of the cilia and the basal body in the IFT46 RNAi cells fed for 16 and 24 h. Some cilia had disorganised microtubules in IFT46-depleted cells (Fig. 3), which likely corresponded to those detected under a fluorescence microscope. Overall, the loss of cilia in IFT80-knockdown cells can be considered a “dilution” process after feeding, whereas IFT46-knockdown cells showed a dramatic decrease in both the length and number of cilia.

**Figure 4:**
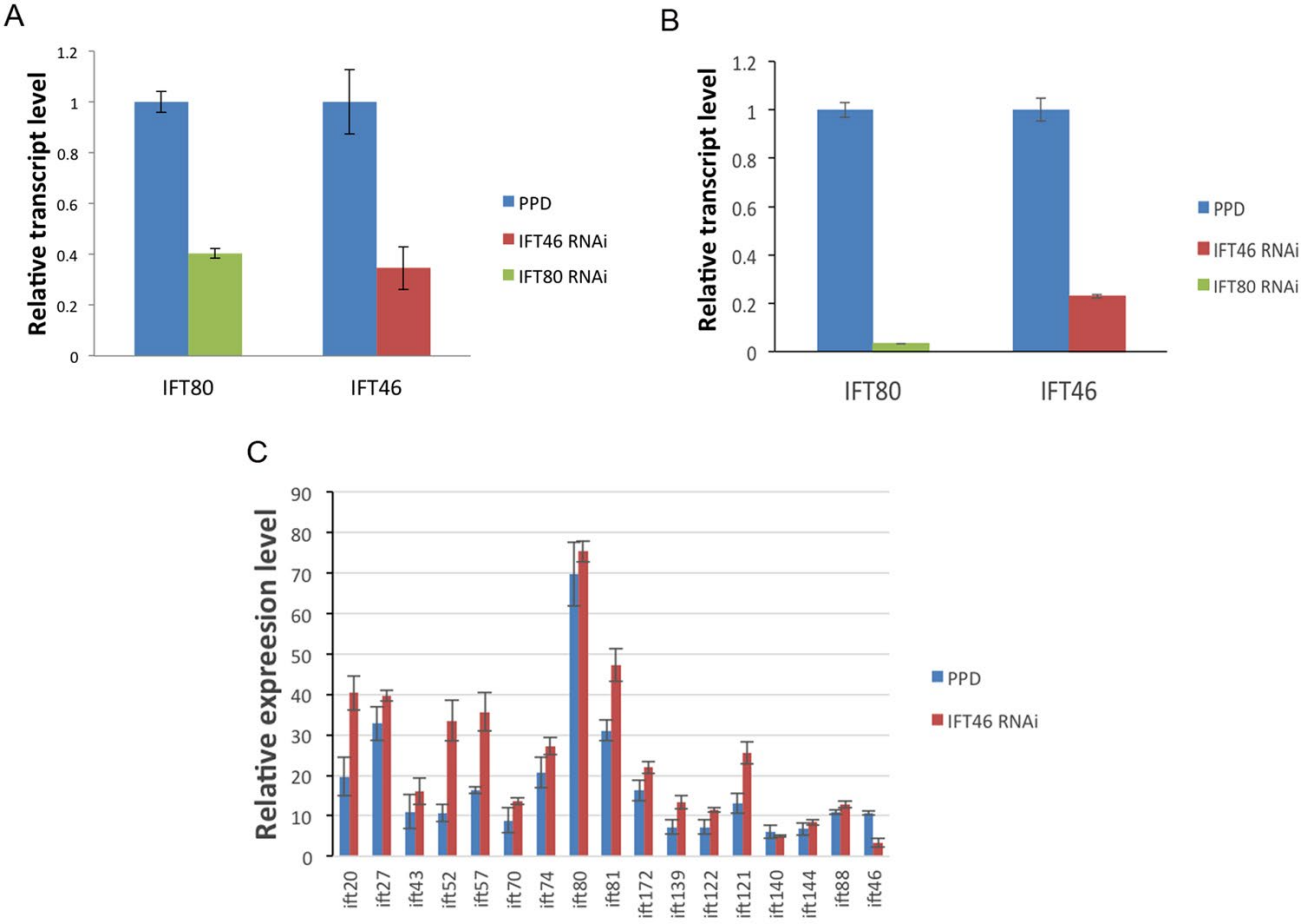
Whole-genome transcriptomic analysis and RT-qPCR confirmed the efficiency of RNAi methods. The transcription level of IFT46 and IFT80 decreased in the group of RNAi in comparison with control group (empty PPD vector). (**A**) High throughput RNA seq showed that the expression level of IFT46 is significant decreased in the group of IFT46 RNAi, as well as IFT80 in the group of IFT80 RNAi. (**B**) Using the expression of tubulin as control, the result of RT-qPCR also showed that the expression of IFT46 and IFT80 are significantly decreased in RNAi group. (**C**) High throughput RNA seq showed the expression level of different IFT proteins in the group of IFT46 RNAi.

Finally, RT-qPCR was performed to detect the expression level of IFT46 and IFT80 in RNAi and control group. We found the expression of IFT46 is significant decreased in IFT46 RNAi cells for 24 h, as well as IFT80 in IFT80 RNAi cells. This result, together with high-throughput RNA seq (three biological replicates) that we did later, confirmed the efficiency of RNAi methods that used in *Paramecium* (Fig. 4A,B). Besides that, using RNA seq, we also found the expression level of most IFT proteins did not change significantly in IFT46 RNAi group, IFT20, IFT52 and IFT57 showed an obviously decrease in IFT46 RNAi cells, that may related to their functions by discoveries before^23–25^ (Fig. 4C).

### GFP fusion experiments show that IFT46 is localised in the basal body and growing cilia of *Paramecium*

**Figure 5:**
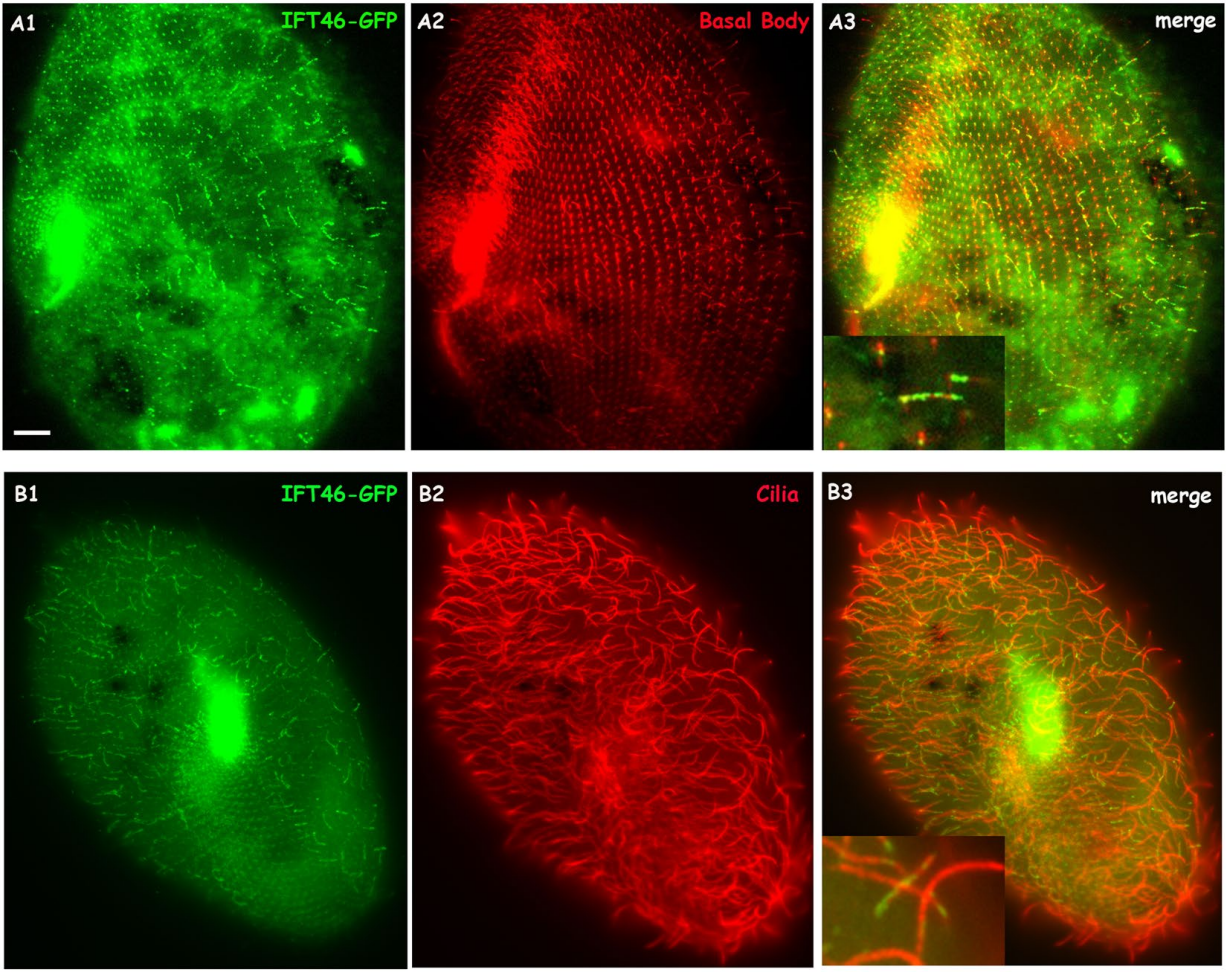
Co-localisation of the IFT46-GFP fusion protein on basal bodies and cilia is displayed by using ID5 and anti-polyglycylated tubulin antibodies, respectively, as markers. (**A1**,**B1**) Green fluorescence displays the localisation of the IFT-46 fusion protein. (**A2**) Basal bodies stained by the ID5 antibody against polyglutamylated tubulin. (**B2**) Cilia labelled by anti-polyglycylated tubulin antibody. (**A3**,**B3**) Merged photos show co-localisation of the IFT46-GFP fusion protein on basal bodies and cilia, and magnifications show details in cilia and basal bodies. Bar: 10 µm.

**Figure 6:**
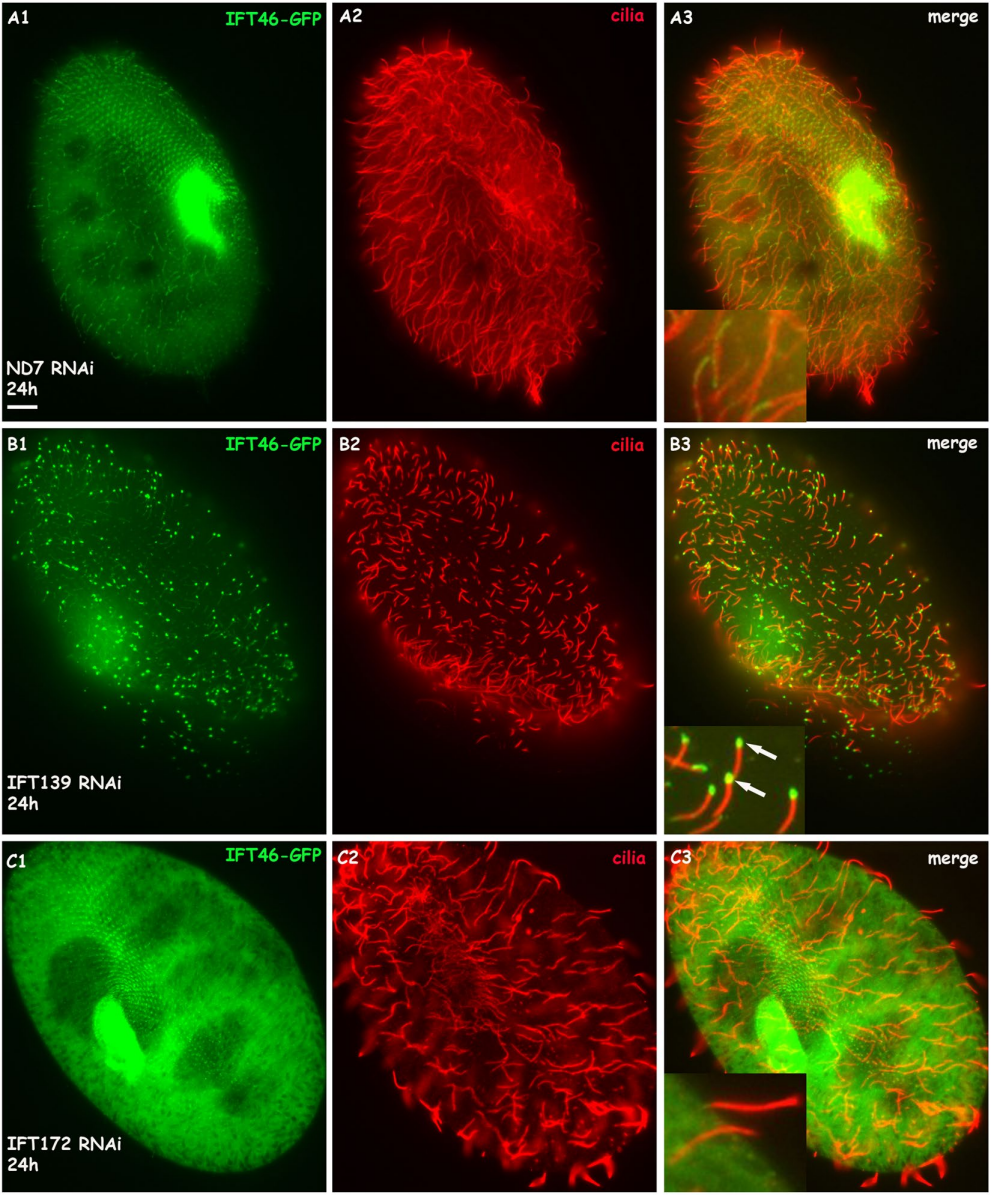
IFT46-GFP fusion protein accumulates at the cilia tip when cells are depleted of IFT139 (IFTA protein) for 24 h but stop entering the cilia when cells are depleted of IFT172 (IFTB protein) for 24 h. (**A1**–**A3**) IFT46-GFP fusion protein localised at basal bodies and some cilia in cells treated with ND7 RNAi as a control. (**B1**–**B3**) IFT46-GFP fusion protein accumulated at the cilia tip of 139 RNAi-treated cells, as shown with arrows in (**B3**). (**C1**–**C3**) IFT46-GFP fusion protein did not localise at the cilia, as shown in (**C3**). Bar: 10 µm.

**Figure 7:**
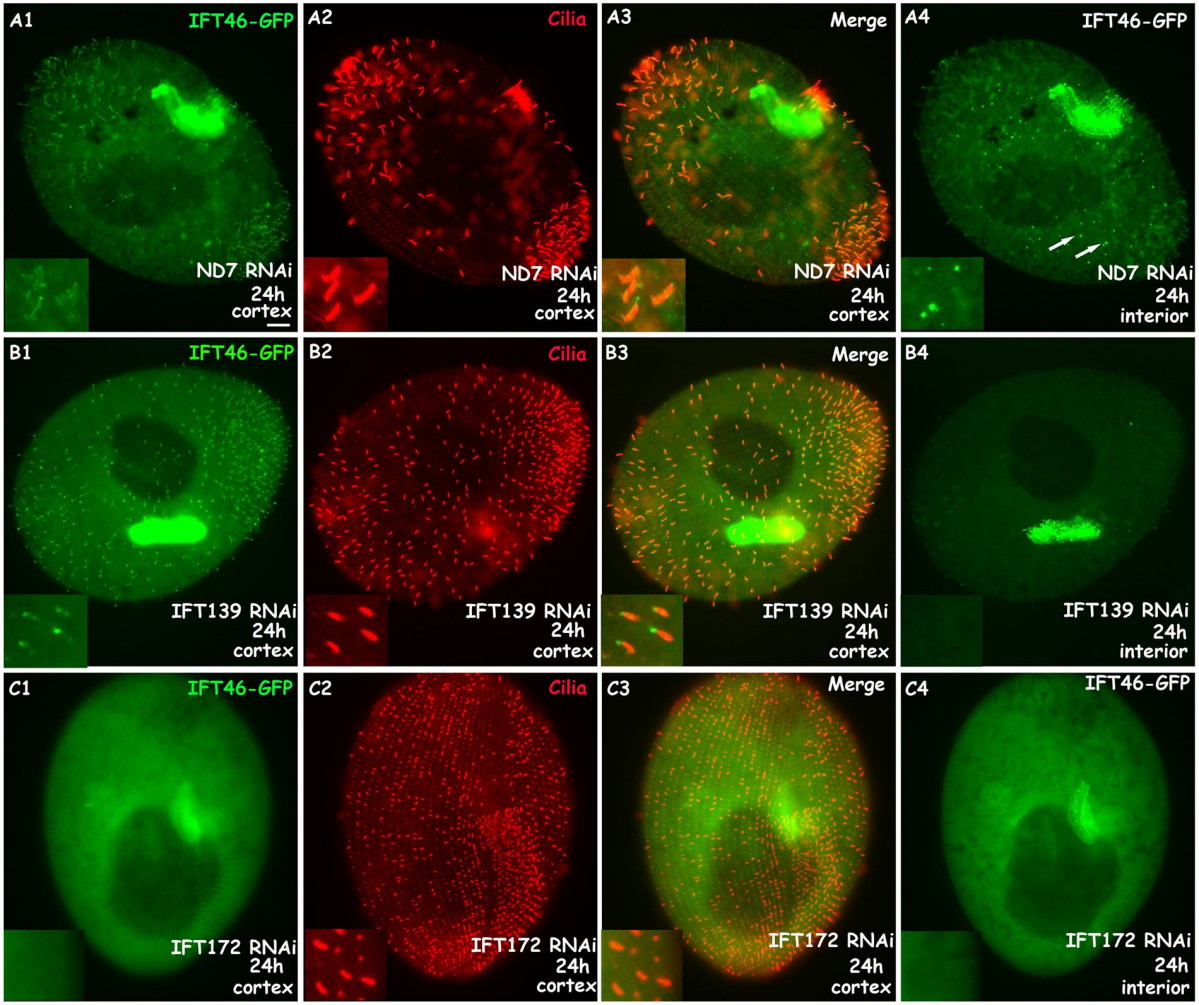
IFT46-GFP fusion protein localised mainly in re-growing cilia and accumulated in the cytoplasm when cells were treated with deciliation for 30 min. (**A1**–**A4**) IFT46-GFP fusion protein localised strongly in the re-growing cilia and cytoplasm of control ND7 RNAi cells after deciliation for 30 min. Arrows show cytoplasmic accumulation. (**B1**–**B4**) IFT46-GFP fusion protein accumulated at the tip of re-growing cilia but did not localise in the cytoplasm of 139 RNAi cells after deciliation for 30 min. (**C1**–**C4**) IFT46-GFP fusion protein did not localise in the re-growing cilia and cytoplasm of 172 RNAi cells after deciliation for 30 min. Bar: 10 µm.

To detect the localisation of IFT46 by expressing the GFP fusion protein in cells, we cloned the IFT46 gene of *Paramecium* with the region containing the putative promoter and the 3′ UTR into the *Paramecium* expression vector pPZZ-GFP02 (see Materials and Methods). Then, the C-terminus of IFT46 fused with GFP was expressed in wild-type *Paramecium* by injecting a sufficient amount of plasmid into the macronucleus of *Paramecium*. The fluorescence was observed in the clonal descendants of the injected cells under a fluorescence microscope. After staining the cilia and the basal body separately by using different anti-tubulin antibodies, we found that the IFT46-GFP fusion protein was localised in the basal body and in some short cilia (Fig. 5). In addition, immunofluorescence photos also show that the depletion of IFT139 (one part of IFT complex A) caused a strong accumulation of the IFT46-GFP fusion protein at the cilia tip, whereas the depletion of IFT172 (one part of IFT complex B) prevented the IFT46-GFP protein from entering the cilia (Fig. 6). To be further, we found that IFT46-GFP displayed stronger fluorescence in all re-growing cilia after the cells were treated by deciliation (see Materials and Methods) (Fig. 7), which indicates that IFT46 may play important roles in cilia assembly. These results agree with the previous results obtained from other models, such as *Chlamydomonas*^19–21^. Moreover, the IFT46-GFP fusion protein acted as one type of moving focus that emerged in the cytoplasm during cilia re-growth after *Paramecium* cells were treated by deciliation. However, we did not observe this cytoplasmic accumulation of IFT46-GFP when *Paramecium* cells were depleted of IFT139 or IFT172 before being treated by deciliation (Fig. 7).

### Depletion of IFT46 provokes an abnormal accumulation of other IFTB proteins in the cortex and cytoplasm of *Paramecium*

**Figure 8:**
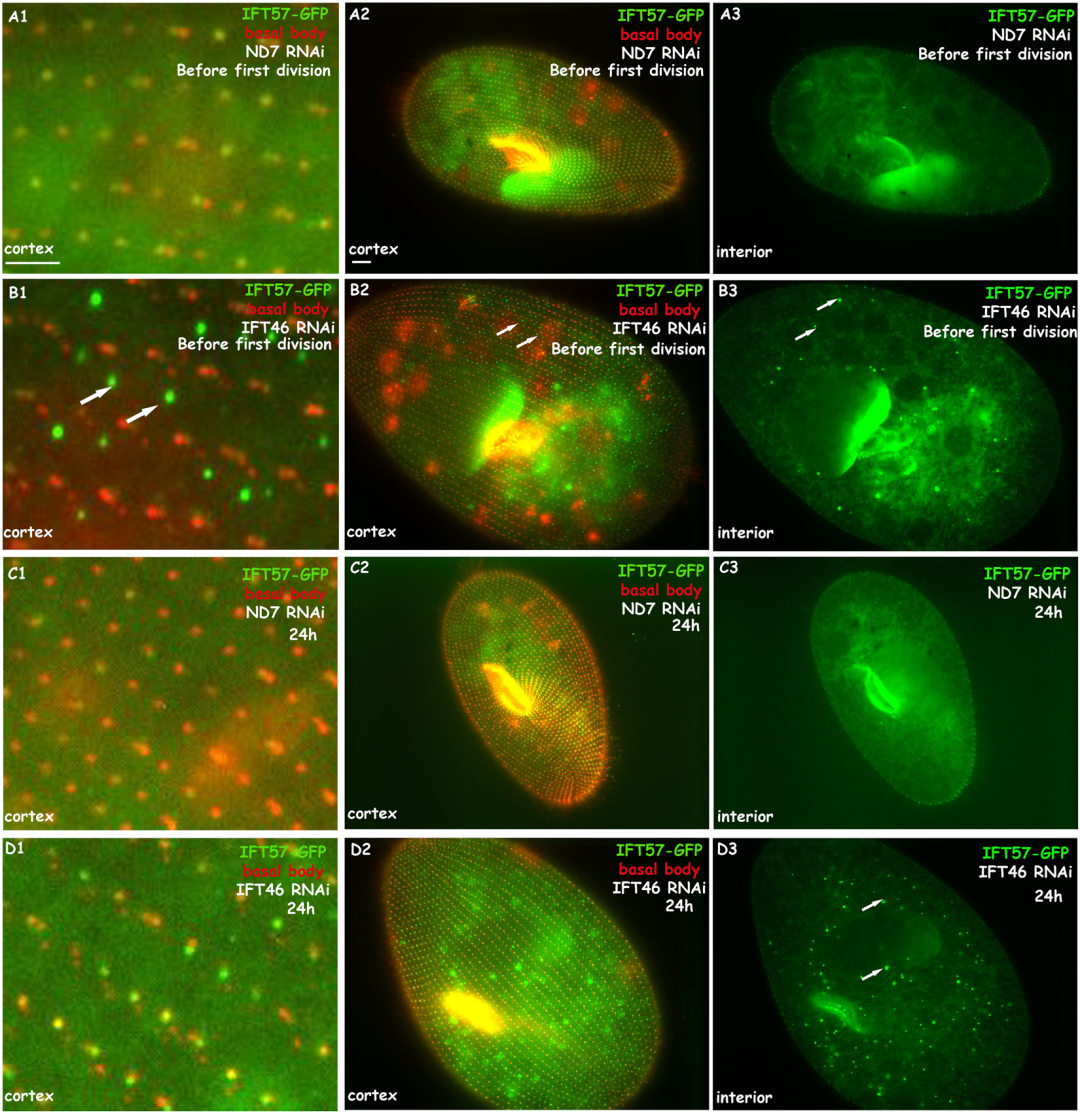
IFT57-GFP fusion protein accumulates in the cortex and cytoplasm when cells are depleted of IFT46. (**A1**–**A3**) FT57-GFP fusion protein localised at basal bodies in cells treated with ND7 RNAi as a control before the first division. (**B1**–**B3**) IFT57-GFP fusion protein accumulated abnormally between basal bodies and in the cytoplasm of IFT46 RNAi cells before the first division, as shown with arrows. (**C1**–**C3**) IFT57-GFP fusion protein localised at basal bodies in cells treated with ND7 RNAi as a control for 24 h. (**D1**–**D3**) Accumulation of IFT57-GFP focused on the cytoplasm of IFT46 RNAi cells for 24 h, as shown with arrows. Bar: 10 µm.

To detect any linking of different IFT proteins in *Paramecium*, we first expressed IFT57-GFP fusion protein in *Paramecium* by microinjection. In our previous study, IFT57, another IFT B member, showed a similar localisation as IFT46 in the basal body and growing cilia in *Paramecium*^14^. Here, using an IFT57-GFP fusion protein expression clone as a control, we silenced the gene of IFT46 in this GFP expression clone and observed the phenotype after feeding. The defects of cilia growth and architecture were as above. Interestingly, an abnormal accumulation of IFT 57-GFP fusion protein was found in the cortex and cytoplasm in the early stage before the feeding cells completed the first division. The cortex accumulation was one type of fluorescent focus, which localised in an orderly manner between the rows of basal bodies (Fig. 8). This IFT57-GFP accumulation was much more focused in the cytoplasm than it was in the cortex after feeding for 24 h (Fig. 8), when IFT46 RNAi cells lost the majority of cilia (Fig. S1). A similar situation was found when qilin-GFP, another representative IFTB, was expressed in IFT46 RNAi cells (data not shown).

### Regulation of *Paramecium* genes by depletion of IFT46

**Figure 9:**
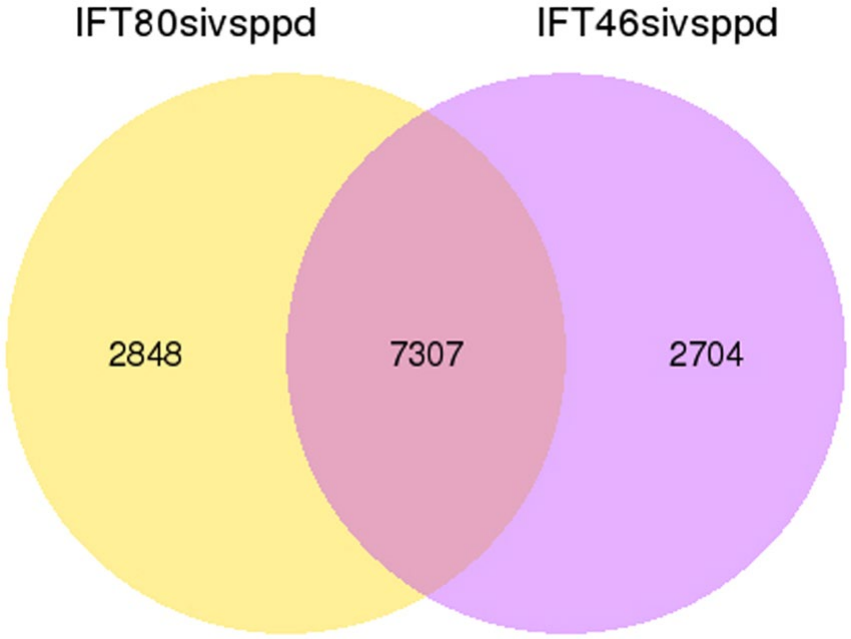
Venn diagram showing the overlap of gene candidates that were differentially expressed after depletion of IFT46 and IFT80. In the Venn diagram, yellow represents the 2848 genes that were differentially expressed after the depletion of IFT80 versus that of IFT46, purple represents the 2704 genes that were differentially expressed by IFT46 knockdown, and the overlap of two circular graphs represents 7303 genes whose expression levels were regulated by both IFT46 and IFT80.

**Figure 10:**
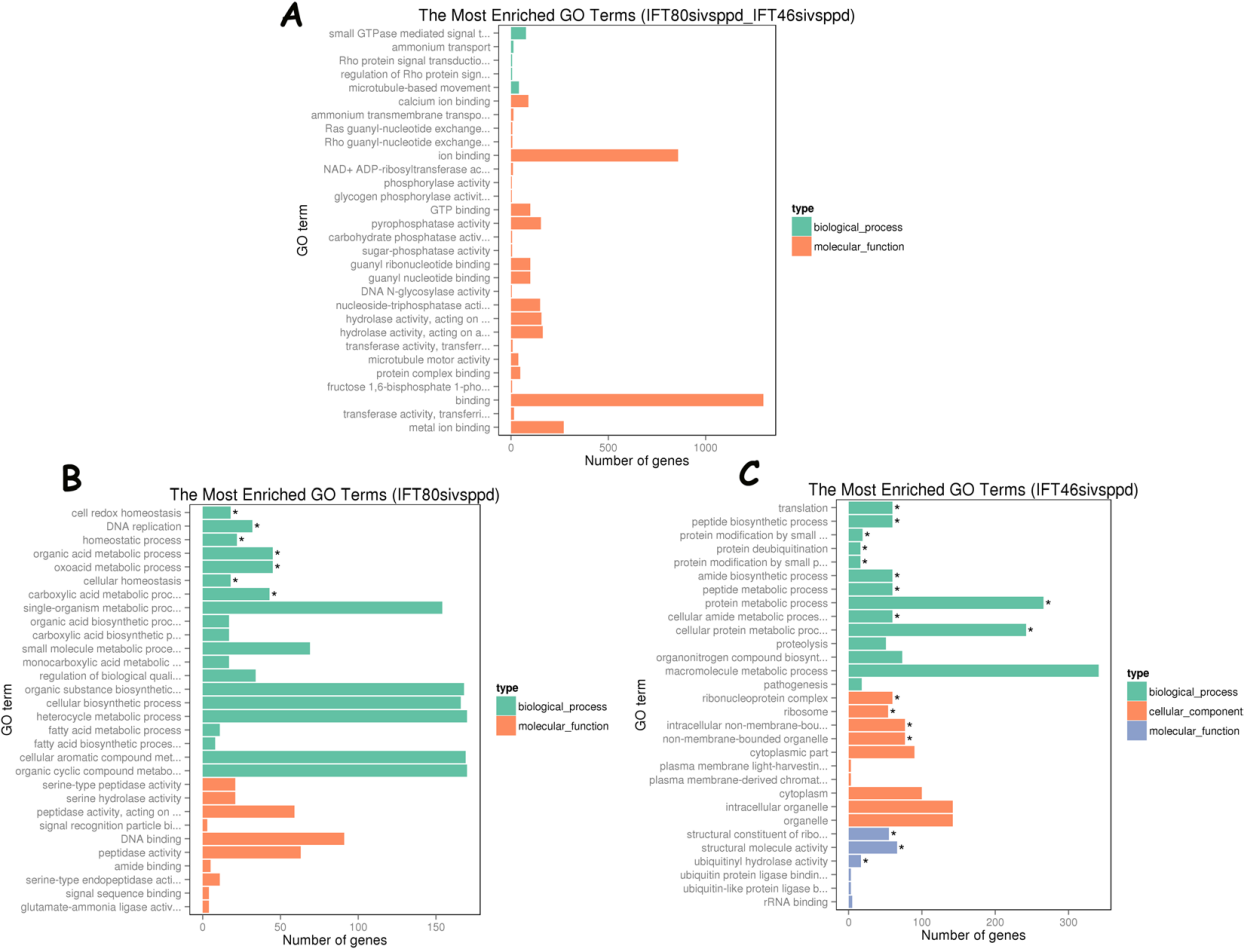
GO enrichment analysis of the gene families in the *Paramecium* genome whose expression was significantly altered by the depletion of IFT46, IFT80, or both. Diagram (**A**) shows the GO categories of most genes that were regulated by both IFT46 and IFT80. Diagram (**B**) shows the GO categories of most genes that were specifically regulated by IFT80. Diagram (**B**) shows the GO categories of most genes that were specifically regulated by IFT46. GO categories include biological process, cell component and molecular function.

The absence of IFT46 and IFT80 in *Paramecium* cells induces different cilia defects, and IFT46 is important for trafficking IFTB proteins between the cytoplasm and the cilia. In the present study, we performed transcriptomic analysis of IFT46 RNAi, IFT80 RNAi and control cells (treated with empty vector) by using the Illumina RNA-seq system. Nine Illumina sequencing libraries from three biological replicates were constructed to analyse the potential changes in gene expression in IFT46 RNAi cells and IFT80 RNAi cells. After filtering, approximately 50 million clean reads were obtained from each of the nine libraries. With the criterion of a 1.5-fold cutoff and significance defined as a P-value < 0.05, differentially expressed genes were assigned to groups through relative analysis and comparison. There were 10,155 genes whose expression level changed in IFT80 RNAi cells compared to the control, including 5075 up-regulated genes and 4980 down-regulated genes. In IFT46 RNAi cells, 10,011 gene candidates were differentially expressed. Among them, 5492 genes were down-regulated, and 4519 were up-regulated. We found that 7307 gene candidates were modulated by both IFT46 and IFT80, 2848 gene candidates were specifically controlled by IFT80, and 2704 gene candidates were specifically regulated by IFT46 (Fig. 9). After the Gene ontology (GO) enrichment analysis of differentially expressed genes, we listed the 30 most significant GO terms for each group, representing different parts of Fig. 10. First, we found that most common genes in the overlapping part of Fig. 10 focused on ion binding and microtubule motor activity under the category of molecular function and on small GTPase-mediated signal transduction, ammonium transport, microtubule-based movement, and Ras and Rho guanyl-nucleotide exchange factor activity under the category of biological process (Fig. 10A, Table S1). We also found that the 2704 gene candidates that were specifically regulated by IFT46 could largely be sorted into structural constituents of ribosomes, structural molecule activity, and ubiquitinyl hydrolase activity under the category of molecular function; peptide biosynthetic process, metabolic process and translation under the category of biological process; and ribonucleoprotein complex, ribosome, intracellular non-membrane-bounded organelle, non-membrane-bounded organelle, cytoplasmic part and cytoplasm under the category of cellular component (Fig. 10C, Table S2. In contrast, the 2848 genes specifically controlled by IFT80 were grouped into cell redox homeostasis, DNA replication, homeostatic process, organic acid metabolic process and oxoacid metabolic process under the category of biological process and into serine-type peptidase activity, signal recognition particle binding, DNA binding and regulation of biological quality under the category of molecular function (Fig. 10B, Table S3).

## Discussion

Since it was first discovered in *Chlamydomonas*, IFT has been confirmed to exist in most eukaryotic cilia and flagella^4^. A previous study revealed that the IFT system is composed of IFTB and IFTA. The former complex, which includes at least 15 members, is linked to a kinesin motor and participates in anterograde transport from the ciliary base to the ciliary tip during cilia building. The latter complex, which contains at least 6 members, is connected to a dynein motor and is responsible for retrograde transport from the ciliary tip to the ciliary base during ciliary recycling. Different IFT proteins play various roles in the IFT complexes. For example, IFT20 is associated with the Golgi apparatus and is linked directly to a kinesin II motor^23,26^. IFT57 stabilises the assembled IFT complex and mediates the transport of motility-related flagellar cargo^24^.

Using different technologies, we confirmed a conserved IFT system in *Paramecium*, a unicellular model with many cilia. In our previous study, IFT57 showed a similar localisation in the growing cilia and the basal body. We found that the depletion of IFT proteins, including IFT139 (a member of IFTA), IFT172, and IFT57 (members of IFTB), provokes defects in cilia growth in *Paramecium*. We also illustrated the dual roles of IFT57 in the cilia and nuclei of *Paramecium*^14^.

The results of this study indicate that different IFT proteins play various roles in cilia building or recycling. IFT80 knockdown in *Paramecium* provoked a marked cilia loss during cell division after RNAi treatment. Most of the remaining cilia of IFT80 RNAi cells displayed a normal length, whereas the depletion of IFT46 caused a dramatic change in both the number and length of cilia after cells were fed bacteria containing the RNAi plasmid. Most cilia in descendant cells after IFT46 RNAi shared lengths between 3 and 8 μm, which were different from those of the control and IFT80 RNAi groups. In addition, IFT46 depletion caused a slower division rate and stronger lethality than did IFT80 knockdown in *Paramecium*. EM photos showed microtubule defects in the IFT46RNAi cells. The IFT46-GFP fusion protein localised in growing cilia and basal bodies, and this IFT46-GFP protein stopped entering the cilia in IFTB RNAi (IFT57 and IFT172 as representatives) cells and became stuck at the cilia tip in IFTA RNAi (IFT139 as a representative) cells. These results suggest that IFT46 plays a conserved role in the IFT system of cilia in *Paramecium*, which is in accordance with the results obtained for other organisms, such as *Chlamydomonas*. Notably, the IFT46-GFP fusion protein accumulated in the cytoplasm when *Paramecium* cells lost almost all old cilia by deciliation, and the cells built new and re-growing cilia. However, this accumulation of IFT46-GFP fusion protein in the cytoplasm was no longer observed in IFTB RNAi or IFTA RNAi cells, suggesting that the putative cytoplasmic function of IFT46 is regulated by other IFT proteins. These results indicate that IFT46 may play a vital and complex role in cilia growth, a process based on vesicle transport.

IFT46 is essential for cilia growth as part of the IFTB complex. In general, IFT46 mutants in green algae, fruit flies and mice display short cilia and defective ciliary structures^19–21^. An intriguing study also showed that IFT46 is required for the transport of ODAs in the cilia of *Chlamydomonas*, as ODA subunits are confined in the cytoplasm outside the ciliary environment in IFT46 mutants. That result suggests that IFT46 carries cargo such as ODAs during ciliary building^14^. Further studies revealed that IFT46 mediates the transport of ODA by binding its cargo-adaptor ODA16 and noted that the C-terminus of IFT46 is important for the interaction with ODA 16^27,28^. The localisation of IFT46 in the basal body and cilia required the association of IFT52^25^. The ciliary entry of the kinesin motor KIF17 depends on its binding to the IFT complex B through IFT46 and IFT56, indicating that IFT46 controls the IFT complexes by entering the cilia^29^.

**Figure 11:**
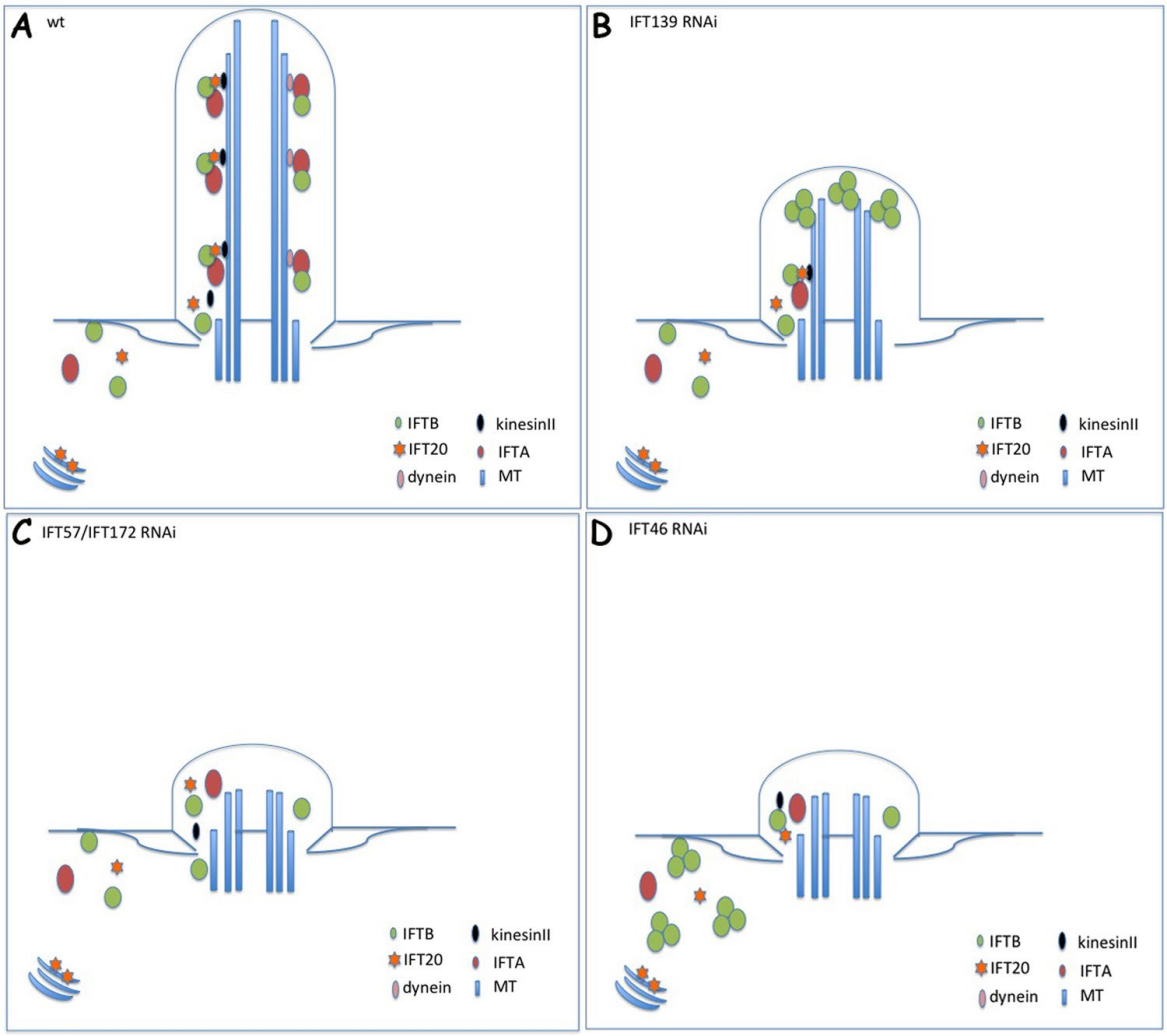
Model illustrating the localisation changes of IFT (**B**) members under knockdown of different IFT genes in *Paramecium*. (**A**) Normal IFT process in WT cells. (**B**) When IFT process was disrupted by the depletion of IFTA proteins such as IFT139, IFTB proteins (IFT46, IFT57) were accumulated at the cilia tip. (**C**) Depletion of several IFTB proteins, including IFT57 and IFT172, prevented other IFTB proteins (IFT46, IFT57) from entering the cilia. (**D**) Absence of IFT46 in *Paramecium* cells provoked the accumulation of other IFTB member (IFT57) in the cortex and cytoplasm.

In the present study, experiments with the IFT57-GFP expression clone showed that the depletion of IFT46 in *Paramecium* initially provoked an aberrant accumulation of the IFT57-GFP fusion protein in the cortex and then became focused in the cytoplasm. Interestingly, this type of accumulation in the cortex sits regularly between two rows of basal bodies, and this phenotype was observed when qilin-GFP expression clone was used as another IFTB candidate (data not shown). Together with the phenomenon that the IFT46-GFP fusion protein accumulated in the cytoplasm during reciliation, our findings suggest that IFT46 plays important roles in the transport of IFT proteins from the cytoplasm to cilia. We have proposed a model to depict these phenotypes (Fig. 11). The model illustrates the characteristic roles of individual IFT members inferred by knocking down different IFT proteins in *Paramecium*.

Based on the above phenomena, we suppose that there might exist a special structure for docking IFT proteins in the cortex of *Paramecium* that recruits IFT members before entering the cilia or after travelling from the cilia tip to the cilia base. When the process is interrupted by the depletion of IFT46, other IFT proteins become stuck in the cortex and cytoplasm of *Paramecium*. However, additional details about this mechanism need to be clarified in further studies. Overall, our findings suggest that IFT46 plays important roles in trafficking IFT proteins between the cytoplasm and cilia in *Paramecium*.

A previous study revealed that IFT46 knockdown in mouse chondrocytes up-regulates selective genes, including *Msx1*, *Fgfr1*, *Col12a*, *Bgn*, *Mmp10* and *Hsp47*, which are involved in growth plate physiology. That study also showed that depletion of IFT46 affects early development in zebrafish^18^. These findings indicate the complex roles of IFT46 in vertebrates. In the present study, we performed a series of high-throughput RNA-seq experiments to detect the potential changes in gene expression caused by the depletion of IFT46 in comparison with a PPD group treated with an empty vector to better understand the function of IFT46 in *Paramecium*. We also checked the profile of transcriptomic expression in IFT80 RNAi cells by RNA-seq, which provided us with a chance to re-examine the roles of two IFTB proteins in one complex at the transcriptome level.

Interestingly, approximately 70% (7307 of 10,011) of the misregulated genes in IFT46 RNAi cells were also affected by IFT80 RNAi. GO enrichment analysis showed that these common genes were concentrated in ion binding and microtubule motor activity under the category of molecular function and in microtubule-based movement under the category of biological process (Fig. 11A, Table S3). Among these, we found that a group of genes that encoded ciliary proteins was regulated by the depletion of IFT46 or IFT80, including dynein heavy chain proteins (DHC7, DHC9), kinesin, kinesin-like protein, and FLA10. These proteins are relevant to the classic roles of IFT80 and IFT46 in cilia or flagella, as previously shown in other organisms. We also found that 2704 gene candidates that were specifically regulated by IFT46 were largely enriched in organelles, intracellular organelles and cytoplasm, which corresponds to the proposition in this study that IFT46 plays specific roles in the cytoplasm and cortex of *Paramecium* cells.

## Methods

### Strains and culture conditions

The wild-type background in this study was represented by the strain of *Paramecium tetraurelia* Stocks d4-2. *P*. *tetraurelia* non-discharge mutant nd7-1, which carries a recessive monogenic mutation preventing trichocyst exocytosis, a dispensable function under laboratory conditions, was used as a recipient in microinjection experiments. *Paramecium* cells were grown at 27 °C in wheat grass infusion BHB (GSE-Vertrieb GmbH, Saarbrücken, Germany), inoculated with naturally ampicillin-resistant bacteria *Klebsiella pneumoniae* and supplemented with 0.8 μg/mL β-sitosterol before use.

### Construction of GFP expression vectors

To detect the cellular localisation of IFT46 in *Paramecium*, a unique IFT46 gene was found by BLAST in *P*. *tetraurelia* from ParameciumDB and shared the ID GSPATG00024708001^30,31^. This gene was cloned with the region containing the putative promoter and the 3′ UTR, which were then cloned into the *Paramecium* expression vector pPZZ-GFP02, a homemade derivative of PPXV-GFP. The putative promoter and the whole gene were amplified, and the green fluorescent protein (GFP) sequence was fused to the 3′ sequence of the gene. The IFT46 gene and its putative promoter were amplified by PCR with primers P1 and P2 (Table S4), then cloned into the region between the *Bam*HI and *Sph*I sites of pPZZ-GFP02. The 3′ UTR of IFT46 was amplified from *Paramecium* DNA with primers P3 and P4 (Table S4) by PCR and then cloned between the *Kpn*I and *Sma*I sites of the pZZ-GFP02 vector. The IFT57 GFP expression vector was from a previous study^14^, which was also based on the *Paramecium* expression vector pPZZ-GFP02.

### Transfection of *Paramecium* by microinjection

Using the a previously described method as described before^32^, a mount of DNA at 5 μg/µL containing a mixture of the plasmids of interest genes and another plasmid containing the control ND7 gene at a ratio of 10:1 was injected into the macronucleus of nd7-1 cells. Microinjection was performed under a Nikon phase-contrast inverted microscope, with a Narishige micromanipulation device and an Eppendorf air- pressure microinjector. Successfully transformed cells were screened for their ability to discharge trichocysts upon picric acid stimulation and were chosen for further study.

### Construction of RNAi vectors

The sequences of genes to be silenced were amplified by PCR and cloned between suitable sites of the polylinker between two T7 promoters of the feeding vector L4440. For the RNAi depletion of IFT46, the DNA sequence of IFT46 from positions 199 to 793 was amplified by PCR with primers P5 and P6, then cloned into the *Spe*I and *Xho*I sites of L4440. For the depletion of IFT80, the DNA sequence of IFT80 from positions 1036 to 1956 was amplified by PCR with the primers P7 and P8, then cloned into the *Kpn*I and *Xho*I sites of L4440. The RNAi vectors that contained gene fragments of IFT172 and IFT139 that we used to silence IFT172 and IFT139 were from Jean and France Koll in Institute for Integrative Biology of the Cell (I2BC), Gif sur Yvette, France.

### RNAi feeding method

RNAi gene knockdown was performed following the method described by Galvani and Sperling^33^. HT115, an RNase III-deficient strain of *Escherichia coli* with an isopropyl-β-D-thiogalactopyranoside (IPTG)-inducible T7 polymerase, was transformed by the desired RNAi vector. Wild-type *Paramecium* cells were incubated with double-stranded-RNA-expressing bacteria and were transferred daily into fresh feeding medium. Control cells were fed bacteria expressing double-stranded RNA corresponding to the complete coding region of the ND7 gene.

To measure the phenotypes induced by RNAi, the length and number of cilia in the control and RNAi groups were recorded separately. *Paramecium* cells were labelled by immunofluorescence with anti-*Paramecium* ciliary tubulin. Then, one cortex focus of a cell was chosen to measure the number and length of all the cilia using ImageJ 1.46 software. Five cells were counted each time, and the experiment was repeated over three times.

### Re-growth of cilia induced by deciliation

To remove cilia from *Paramecium* cells and follow ciliary growth during reciliation, a few hundred cells were first collected in a small volume and then transferred to a 1.5 mL microtube containing 1 mL of 5 μL ethanol and 1 mM CaCl_2_. After 30 s of vortexing and 15 s of centrifugation, the cells were placed back into fresh medium at 27 °C and then collected at various designed times.

### Immunofluorescence microscopy

Immunostaining of cells was carried out as previously described using the anti-*Paramecium* ciliary tubulin at a 1:400 dilution^34^ and TAP952 antibody against monoglycylated tubulin (Millipore) at a 1:80 dilution to label cilia. The basal body was labelled with the monoclonal ID5 antibody against polyglutamylated tubulin at a 1:50 dilution. Appropriate anti-rabbit IgG and anti-mouse IgG fluorescent secondary antibodies were applied at 1:500 dilution (Sigma). GFP-fusion cells were initially fixed in paraformaldehyde for 30 s and then treated with 0.5% Triton-X100 in PBS buffer for 30 min. Nuclei were stained with Hoechst-33258, and slides were mounted in Cilifluo (London). The preparations were observed under a Zeiss Axioskop 2-plus fluorescence microscope equipped with a Roper Coolsnap-CF intensifying camera with GFP, TRITC and DAPI filters. Images were processed with Metamorph software (Universal Imaging). Alternatively, samples were also observed under a Confocal LEICA TCS SP8 microscope equipped with argon and helium-neon lasers using EZ-C1 3.30 software for acquisitions.

### Transmission electron microscopy (TEM)

TEM was performed as previously described with some modifications^35^, Paramecium cell pellets were prefixed in a 1:1 mixture of 2% OSO_4_ and 2.5% glutaraldehyde at 4 °C for 10 min. The fixed cells were then washed several times with 0.1 M phosphate buffer and post-fixed in 1% OSO_4_ at 4 °C for 1 h. The post-fixed cells were washed again and then dehydrated through a graded series of acetone and embedded with Epon 812. Ultrathin sections were stained with uranyl acetate and lead citrate, then examined with a Hitachi 7700 (100 kV) transmission electron microscope.

### Total RNA extraction

Paramecium cells in RNAi and control groups were collected separately after treatment with bacteria containing RNAi or empty vector for 16 h. Then, the cells were washed in Dryl buffer twice. Subsequently, the total RNA of each group was extracted by using Trizol (Sigma, USA).

### Quantitative PCR

To detect the gene expression level, two micrograms of total RNA were reverse transcribed into cDNA by ImPromII reverse transcriptase (Promega). Real-time PCR was performed with the SYBR Green I Master (Roche) on a LightCycler 480 (Roche). At least two biological replicates and two technical repeats for every biological replicate were tested. And the gene expression level was normalized with that of α-TUBULIN. The primers used in this study are listed in Supplementary Table S4.

### Whole-genome transcriptomic analysis

Nine Illumina sequencing libraries from three biological replicates were constructed using the RNA Library Prep Kit (NEB, USA) in accordance with the manufacturer’s recommendations^36^. In brief, mRNA was purified from total RNA using poly-T oligo-attached magnetic beads. Fragmentation was carried out using divalent cations under elevated temperature in NEBNext First-Strand Synthesis Reaction Buffer (5×). First-strand cDNA was synthesised using random hexamer primers and M-MuLV Reverse Transcriptase (RNase H-). Second-strand cDNA was synthesised using DNA polymerase RNase H. Remaining overhangs and I were converted into blunt ends by exonuclease/polymerase activities. After the adenylation of 3′ ends of DNA fragments, NEBNext Adaptors with a hairpin loop structure were ligated to prepare the fragments for hybridisation. To select cDNA fragments that were preferentially 150–200 bp long, library fragments were purified with the AMPure XP system (Beckman Coulter, Beverly, USA). Then, 3 μL of USER Enzyme (NEB, USA) was used with size-selected, adaptor-ligated cDNA at 37 °C for 15 min followed by 5 min at 95 °C. Then, PCR was performed with Phusion High-Fidelity DNA polymerase, Universal PCR primers and Index (X) Primer. PCR products were purified (AMPure XP system), and library quality was assessed using the Agilent Bioanalyzer 2100 system. After cluster generation, the library preparations were sequenced on an Illumina HiSeq platform, and 125 bp/150 bp paired-end reads were generated. Reference genome and gene model annotation files were downloaded from the genome website directly (http://www.ncbi.nlm.nih.gov/genome/term=Paramecium_tetraurelia). The index of the reference genome was built using Bowtie v2.2.3, and paired-end clean reads were aligned to the reference genome using TopHat v2.0.12. Differential expression analysis of three conditions/groups (three biological replicates per condition) was performed using the DESeq R package (1.18.0). The resulting P-values were adjusted using the Benjamini and Hochberg approach for controlling the false discovery rate. Genes with an adjusted P-value < 0.05 found by DESeq were defined as differentially expressed.

Gene ontology (GO) enrichment analysis of differentially expressed genes was implemented by the GOseq R package^37^, in which gene length bias was corrected. GO terms with corrected P-values < 0.05 were considered significantly enriched by differentially expressed genes.

## Electronic supplementary material

Supplementary Information

Table S1

Table S2

Table S3
