# Supplementary material for: Intraflagellar transport 46 (IFT46) is essential for trafficking IFT proteins between cilia and cytoplasm in Paramecium
Source: Sci Rep. 2018 Jun 18;8:9259. doi: 10.1038/s41598-018-27050-8 (PMC6006156; doi:10.1038/s41598-018-27050-8)
Supplement: Supplementary file 1 — Supplementary Information [file 41598_2018_27050_MOESM1_ESM.pdf]

Supplementary work

Intraflagellar transport 46 (IFT46) is essential for trafficking IFT proteins between cilia and cytoplasm in *Paramecium*

Lei SHI[1]* Xuezhen SHI[2] & Yuan SHEN[3]*

[1]School of Basic Medical Sciences, Xinxiang Medical University, Xinxiang, 453003, P.R. China.
[2]School of Life Science, Henan Normal University, Xinxiang, 453003, P.R. China.
[3]School of Pharmacy, Xinxiang Medical University, Xinxiang, 453002, P.R. China

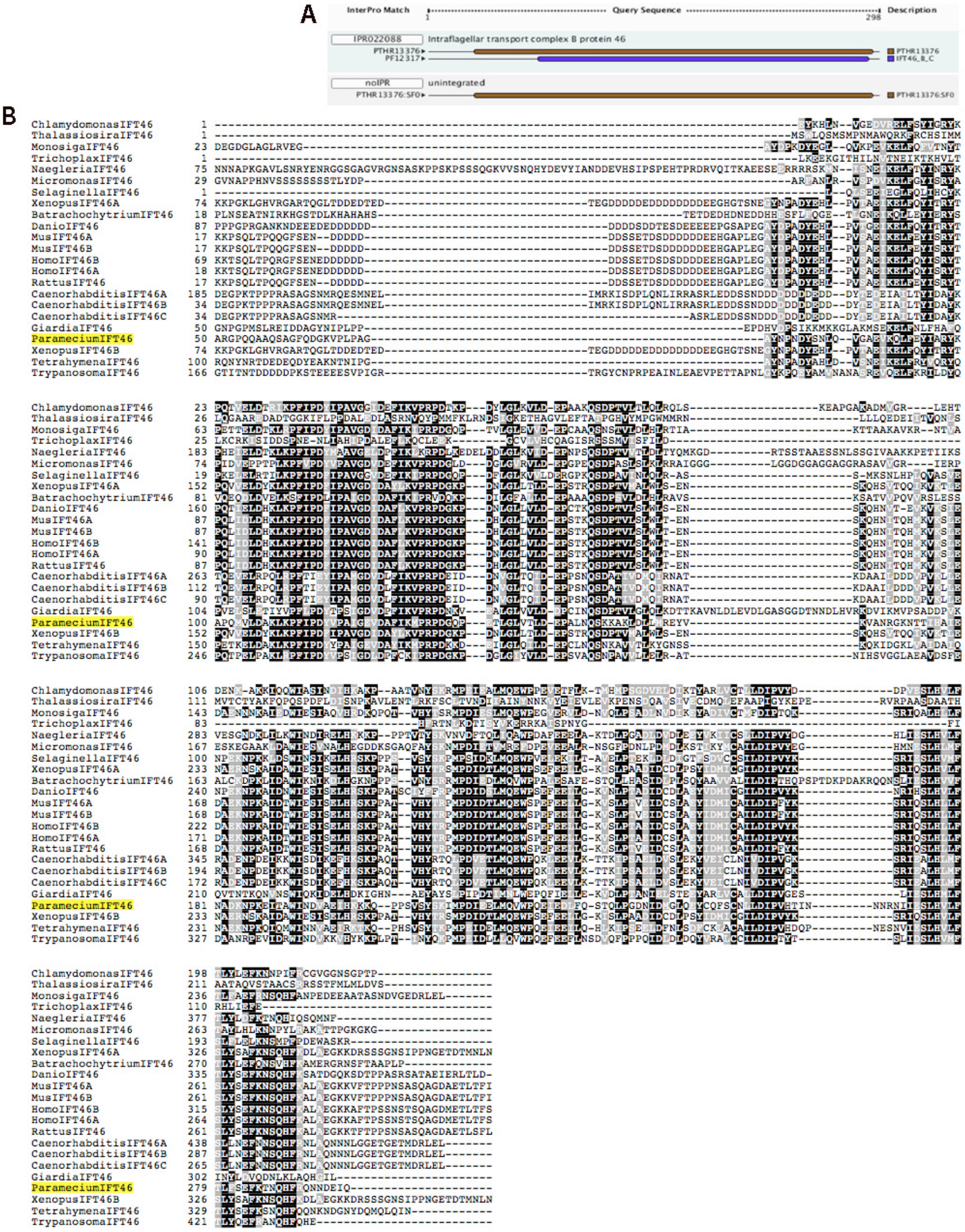

Figure S1. A: InterPro scanning revealed that IFT46 contained a conserved C-terminal domain (IFT46_B_C) **B:** The alignment of IFT46 proteins of various species shows that they are highly conserved through evolution, including in *Paramecium*.

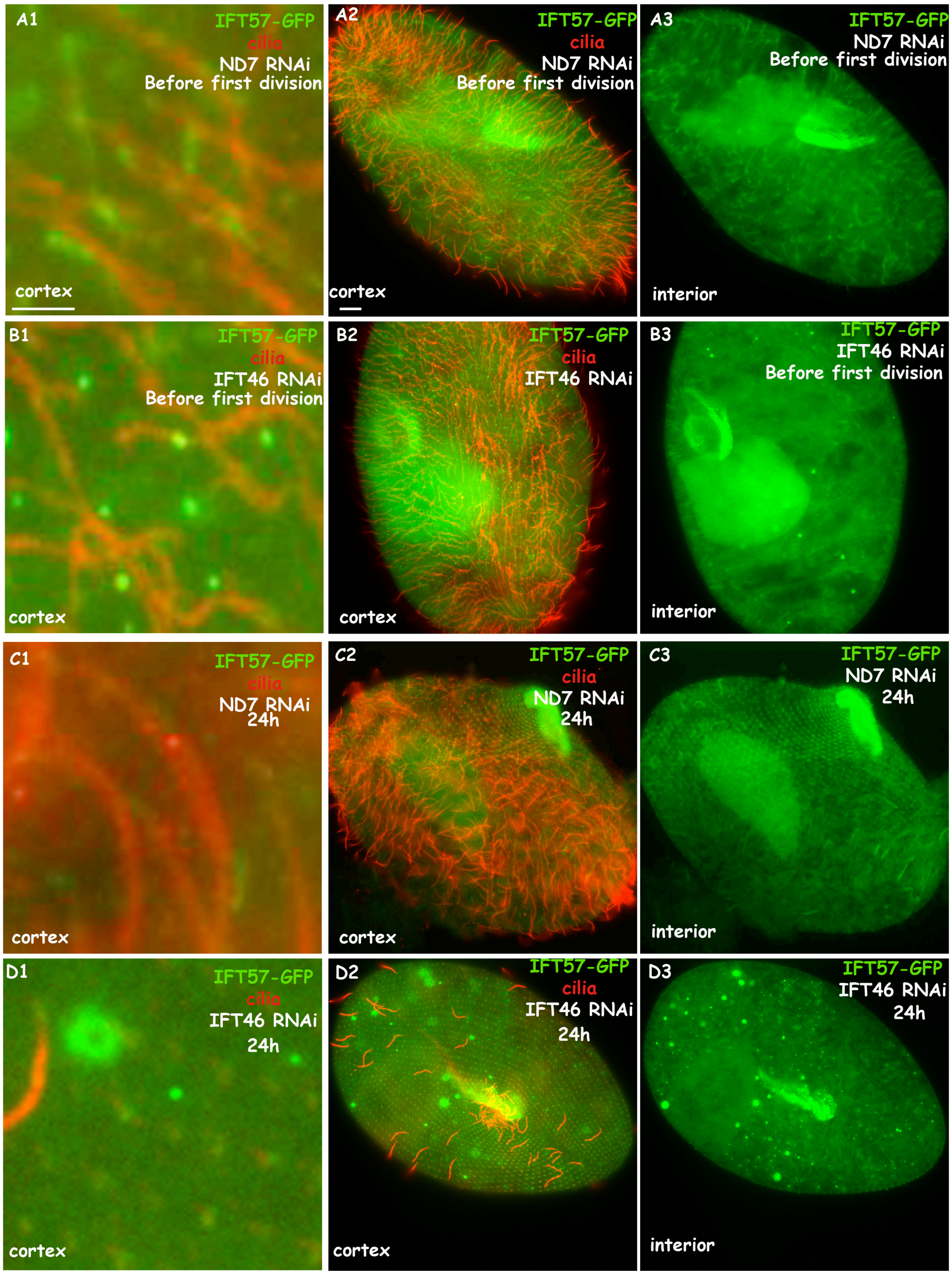

Figure S2. IFT57-GFP fusion protein accumulated at the cortex and cytoplasm when cells lost most cilia after treated with IFT46 RNAi.

A1–A3: IFT57-GFP fusion protein localised at some cilia in cells treated with ND7 RNAi as control before the first division; B1–B3: IFT57-GFP fusion protein accumulated abnormally between basal bodies and in the cytoplasm of IFT46 RNAi-treated cells before the first division; C1–C3: IFT57-GFP fusion protein localised at some cilia in cells treated with ND7 RNAi as control for 24 h; D1–D3: Accumulation of IFT57-GFP focused on the cytoplasm of IFT46 RNAi cells for 24 h, when cell lost most cilia. Bar: 10 µm.

Legend to Supplementary TableS1, TableS2 and TableS3

Table S1. GO analysis lists the richest categories of common genes whose expression are both modified by depletion of IFT46 and depletion of IFT80.

Table S2. GO analysis lists the richest categories of genes whose expression are specifically modified under depletion of IFT46.

Table S3. GO analysis lists the most richest categories of genes whose expression are specifically modified under depletion of IFT80

Primer ID	Primer sequence	Use
P1	5'-atacttggatccTATTTAATATAATTAAATAAATACTGTTATTC -3'	IFT46 (gene)
P2	5'- GACAAAACAATGATGAAATTTAAggcatgcaagtat -3'	IFT46 (gene)
P3	5'- atacttggtacctgaATATTCAATAATATAACATTATTTTC -3'	IFT46 (3'-UTR)
P4	5'- GGAATATTTATTATAATTATTAA -3'	IFT46 (3'-UTR)
P5	5'- ttattactagtGCTGGAGCTAGAGGACCCCAATAAGC -3'	RNAi IFT46
P6	5'- GGTGTGGCCACAAGAGATTGAAGctcgagaacc -3'	RNAi IFT46
P7	5'- ttattCTCGAGCAAATTATTGATTTAATAGATG-3'	RNAi IFT80
P8	5'- ggttGGTACCGACCAGCAAGTGCAGCCCACAATG-3'	RNAi IFT80
P9	5'- AAGTCGGTAATGCCTGTTGG-3'	qPCR α-tubulin
P10	5'- ATTCAAGGCACCATCGAATC-3'	qPCR α-tubulin
P11	5'- CACACAATTACCAGGGGACA-3'	qPCR IFT46
P12	5'- CAGGAATGTCTAATAAATTGCATGA -3'	qPCR IFT46
P13	5'- TGGAGTCCAGACAATGATGG -3'	qPCR IFT80
P14	5'- TCCACCTTCAAAACCAAACC -3'	qPCR IFT80

Table S4. Primers used for the cloning and RT-qPCR of this study.
